# New Potential Pharmacological Options for Endometriosis Associated Pain

**DOI:** 10.3390/ijms25137068

**Published:** 2024-06-27

**Authors:** Laura García-Izquierdo, Pilar Marín-Sánchez, Pilar García-Peñarrubia, María Martínez-Esparza

**Affiliations:** 1 Department of Biochemistry, Molecular Biology (B) and Immunology, Faculty of Medicine, Regional Campus of International Excellence “Campus Mare Nostrum”, University of Murcia and Biomedical Research Institute of Murcia (IMIB), 30120 Murcia, Spain; ml.garciaizquierdo@um.es (L.G.-I.); pigarcia@um.es (P.G.-P.); 2 Department of Gynaecology and Obstetrics, Hospital General Universitario Santa Lucía, University of Murcia and Biomedical Research Institute of Murcia (IMIB), 30120 Murcia, Spain; ibsyna@hotmail.com

**Keywords:** endometriosis, immunotherapy, drugs, pain

## Abstract

Endometriosis is a chronic inflammatory disorder characterized by the abnormal growth of endometrial-like tissue outside the uterine cavity, affecting 10–15% of women of reproductive age. Pain is the most common symptom. Treatment options include surgery, which has limited effectiveness and high recurrence rates, and pharmacotherapy. Hormonal therapies, commonly used for symptom management, can have side effects and contraceptive outcomes, contributing to the infertility associated with endometriosis, with pain and lesions often reappearing after treatment cessation. Among its etiological factors, immunological and inflammatory dysregulation plays a significant role, representing an interesting target for developing new therapeutic strategies. This review critically analyzes recent studies to provide an updated synthesis of ongoing research into potential new pharmacotherapies focusing on lesion progression, pain relief, and improving quality of life. Immunotherapy, natural anti-inflammatory and antioxidant compounds and drug repurposing show promise in addressing the limitations of current treatments by targeting immunological factors, potentially offering non-invasive solutions for managing pain and infertility in endometriosis. Promising results have been obtained from in vitro and animal model studies, but clinical trials are still limited. More effort is needed to translate these findings into clinical practice to effectively reduce disease progression, alleviate pain symptoms and preserve the reproductive capacity, improving patients’ overall wellbeing.

## 1. Introduction

Endometriosis (EM), a chronic inflammatory disorder characterized by abnormal endometrial-like tissue growth beyond the uterine cavity, profoundly impacts 10–15% of reproductive-aged women, though accurately determining its prevalence remains challenging [1,2,3]. The prevalence of EM is reported to be higher in developing countries compared to developed countries [4,5]. Although it is frequently noted as most common among white women, factors such as race, ethnicity, socioeconomic status, and gender can greatly influence access to diagnosis and treatment. These disparities can result in an inaccurate representation of the condition’s true prevalence [6,7]. Symptomatically diverse, it presents with dysmenorrhoea (90% of cases), chronic pelvic pain (CPP, 77%), dyspareunia (76%), dyschezia (66%), and hematochezia, affecting physical, sexual, psychological, and social well-being [8].

**Figure 1:**
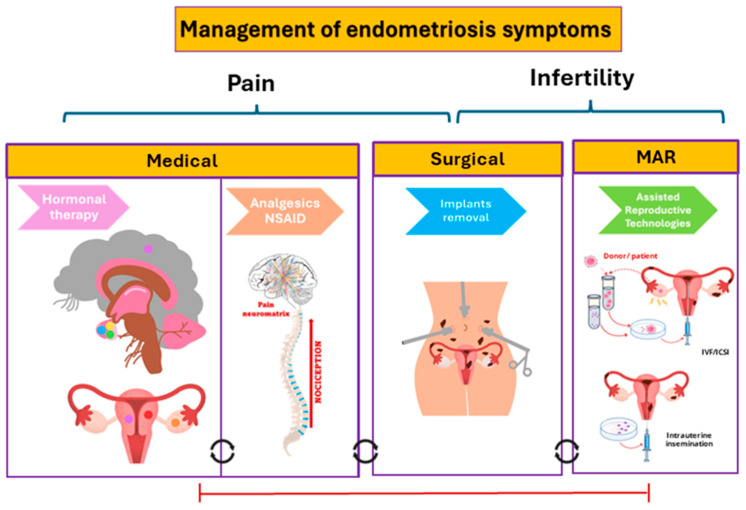
Current options for management of endometriosis symptoms. Pain is the main symptom associated with EM and is currently treated with medical (hormonal therapy, analgesics and NSAID) or surgical strategies. Adverse effects, contraceptive effects, and recurrence, are significant challenges associated with these therapeutic options, often exacerbating the infertility associated to EM. Surgery and medically assisted reproduction (MAR) are options for treating infertility, however, ovarian stimulation and/or cessation of pain management hormone therapy in order to treat infertility can increase the lesion growth and pain. The red line indicates that the options are mutually exclusive, while the circular black lines mean that the options could be combined. IVF, in vitro fertilization; ISCI, Intracytoplasmic Sperm Injection.

The treatment of pain associated to EM (Figure 1) involves surgery or medical therapies [9] While surgery remains a viable option for EM-associated pelvic pain (PP), its efficacy is compromised by drawbacks, including a 40–50% postoperative recurrence at a 5-year follow-up [10]. Surgical interventions like ovarian endometrioma removal and excision of deeply infiltrating forms pose risks, such as diminished ovarian reserve and heightened complications [11].

Current pharmacological interventions include hormonal treatments, analgesics, and non-steroidal anti-inflammatory drugs (NSAID). Hormonal therapy involves the use of medications that impact the levels of sex hormones to reduce the growth and activity of endometrial tissues, to alleviate symptoms such as pain and inflammation. Combined hormonal contraceptives, progestogens, gonadotropin-releasing hormone (GnRH) agonists, GnRH antagonists and aromatase inhibitors are current options for hormonal therapies [7]. Medical treatment for EM typically does not eliminate the disease, often involves side effects and can hinder reproductive desires of patients. CPP associated with EM frequently necessitates repeated courses of medical therapy, surgical intervention, or both, with symptoms commonly recurring once the therapy is stopped [7]. Treatment options for infertility associated with EM include surgery and medically assisted reproduction (MAR), which requires stopping hormonal therapy. In some cases, ovarian stimulation during MAR can exacerbate lesion growth and pain [7]. Therefore, developing new therapeutic strategies is crucial.

Recognizing the challenges of medical and surgical current treatment options described above, there is an urgent need to develop additional therapeutic options that are effective and devoid of side effects. This literature review aims to identify new pharmacotherapies under investigation directed to the EM associated pain, highlighting their potential benefits and the stages of development they are in for possible clinical application. The articles selected for this review, published primarily in the last five years, demonstrate positive results with molecules tested in vitro and in vivo, with animal models, or patients with EM, that can be considered as potential alternatives to currently used treatment, not as adjuvants, focusing on lesion progression, pain relief or improved quality of life (QoL). Clinical trials that have not been completed or whose results have not been published have not been included. Studies involving compounds found ineffective in woman were not included.

Our exploration aims to encourage future research and advances in clinical care by offering an alternative approach to reduce disease progression, alleviate pain symptoms, preserving the reproductive capacity, to improve patients’ overall wellbeing.

## 2. Immunotherapy

A dysregulated state of macrophages and other immune cells, contributes to the maintenance of a chronic inflammatory environment, playing a critical role in the development of endometriotic lesions [12]. Because of the involvement of immune dysregulation in the pathophysiology of EM, immunotherapy could represent a promising and valuable approach for treating this condition.

**Figure 2:**
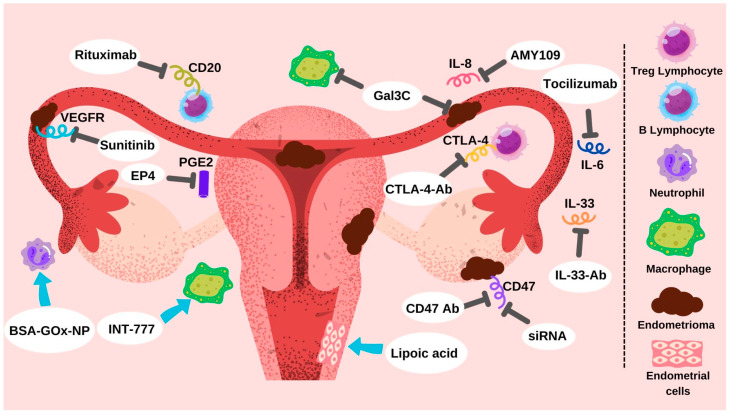
Potential immunotherapy targets in the management of EM. This figure shows the potential targets of immunotherapy in the reproductive tract and abdominal cavity for the management of EM progression and associated pain.

Classical immunotherapy, utilizing monoclonal antibodies, checkpoint inhibitors, and cytokines, initially developed for cancer treatment, has been extended to other pathologies with significant results. Immunotherapeutic compounds under study for EM associated pain treatment are summarizes in Figure 2 and Table 1.

Interleukin 33 (IL-33), a member of the IL-1 family, significantly stimulates the production of T helper-2 (TH2) cytokines, which in turn promote M2 macrophage polarization. Researchers investigated the effects of **IL-33 antibody** and **erastin**, both individually and in combination, in human samples from EM patients and a control group with benign gynaecological conditions, as well as in mouse samples. Erastin promoted the progression of ferroptosis in eESC, whereas the IL-33 antibody reduced the eESC tolerance to ferroptosis and stimulated macrophage polarization towards the pro-inflammatory M1 subtype, thus enhancing immune defences. The IL-33 antibody inhibited the development of EM, and its combination with erastin further amplified this inhibitory effect [13].

Interleukin-8 (IL-8) is a cytokine involved in inflammation and the adhesion/migration of neutrophils and monocytes within the endothelium, potentially leading to macrophage and lymphocyte infiltration in tissues. The therapeutic potential of a long-acting **antibody against IL-8** (AMY109) was evaluated in a cynomolgus monkey model of EM. Monkeys received subcutaneous injections of 2 or 10 mg/kg every 4 weeks for 6 months. This treatment resulted in a reduction in the volume of nodular lesions and adhesions. AMY109 effectively inhibited neutrophil recruitment to endometriotic lesions, reduced the production of monocyte chemoattractant protein-1 by neutrophils, and decreased cynomolgus migration. Additionally, AMY109 did not alter the menstrual cycle. The plasma half-life of AMY109 in monkeys (20 days after administration) was much longer than that of other conventional antibodies, suggesting that monthly subcutaneous administration is feasible. Current studies are evaluating the efficacy and safety of AMY109 in humans [14].

Interleukin- 6 (IL-6) is a cytokine that plays a significant role in the immune system by regulating immune responses, inflammation, and hematopoiesis. The IL-6 can share in the formation of EM by blocking the apoptosis of ectopic implants in the peritoneum. The study by El-Zayadi et al. [15] showed that treatment with an **anti-IL-6 receptor monoclonal antibody** (tocilizumab) significantly reduced the size of endometriotic lesions and decreased the levels of inflammation, in a rat model of EM.

CTLA-4 (Cytotoxic T-Lymphocyte Antigen 4) is a protein receptor located on the cell membranes of T and B cells. Stimulation of the CTLA-4 receptor inhibits lymphocyte function, and its levels are increased in EM [16]. The effect of an **anti-CTLA-4 antibody** (10 µg/mL) encapsulated in poly (lactic-co-glycolic acid) (PLGA) as a protein delivery vehicle was assayed in a mouse model of EM. This treatment targeted the surface of CD4+CD25+ regulatory T cells (Treg cells) and induced a gradual reduction in Treg cell numbers, leading to sustained suppression of both proliferation and invasion of ectopic endometrial cells [17].

CD47 is widely expressed on normal cells and overexpressed on tumour cells. Its binding to SIRPα on the cell membrane of macrophages inhibits phagocytosis and triggers tumour immune escape [18]. Studies using **siRNA** (small interfering RNA) or **neutralizing antibodies against CD47** have shown increased phagocytic capacity of macrophages and promoted apoptosis of ESC in co-cultures with human euESC from the control group, as well as euESC and eESC from the EM group over 24 h [19]. In an in vivo mouse model of EM, 12 h after embryonic stem cells were injected into the abdominal cavity of mice, LPM from CD47 knockout mice showed an increased phagocytic ability and greater efficiency in clearing ectopic endometrial cells compared to wild- type mice [19].

**Rituximab**, a monoclonal antibody against the specific surface protein of B lymphocytes, CD-20, is an immunomodulatory drug used in rheumatoid arthritis and B-cell lymphomas. Rituximab caused a statistically significant regression in the size of the endometriotic implant in a rat model of EM. Treatment with rituximab also induced significant differences in B cell count and fibrosis score compared to the control group [20].

In human peritoneal macrophages from women with EM, PGE2 receptor expression (EP2 and EP4) was elevated. An **EP4 antagonist** reduced cAMP levels and expression of vascular endothelial growth factor, chemokine 2 ligand, and mRNA of chemokine ligand 3. Selective EP2 and EP4 antagonists reduce EP-mediated actions, making them potential therapeutic agents for controlling inflammation and EM-associated pain [21].

**Lipoic acid** (LA) is an endogenously produced small molecule that exhibits antioxidant and anti-inflammatory effects. LA treatment reduced inflammatory phenotype of healthy monocytes and macrophage-derived monocytes, decreasing inflammatory cytokine secretion and phagocytosis via a cAMP-mediated mechanism. Treatment with LA is protective in inflammatory diseases as multiple sclerosis [22]. The study of Di Nicuolo et al. [23] on human endometriotic epithelial and stromal cell line cultures concluded that α-LA significantly reduces the activity of ER-β and the cytoplasmic inflammasome-3, NALP-3, and the secretion IL-1β and IL-18. It reduces cell adhesion and invasion using a 3D culture invasion assay, through decreased expression of ICAM-1 adhesion molecule and matrix-metalloprotease (MMP) 2 and 9 activities. Therefore, LA could inhibit the progression of endometriosis.

The TGR5 (Takeda-G-protein-receptor-5) is a cell surface receptor for bile acids, implicated in the suppression of macrophage functions and regulation of energy homeostasis by bile acids. A study conducted in patients showed that the **synthetic TGR5 agonist** (INT-777) has protective effects against inflammation and reactive oxygen species (ROS) in cytokine-induced activation of human ESC. Therefore, INT-777 may have an implication in the clinical treatment of EM [24].

**Sunitinib** is an oral oxindole multitargeted kinase inhibitor that inhibits certain receptor tyrosine kinases and is used as an anti-cancer drug. In 2022, the effect of sunitinib was studied in women with EM. This study concluded that sunitinib reduces the migration capacity of endometrial cells by involving the p-VEGFR-PI3K-AKT-YBX1-Snail signalling pathway [25].

Gal-3 deficiency and treatment with the **Galectin-3 (Gal-3) inhibitor, Gal3C**, significantly reduced the development of EM as shown in a mouse model treated for 15 days. There was a decrease in the implantation and size of lesions, as well as in the expression of COX-2, TGFβ1 (transforming growth factor β1), VEGF, VEGFR-2, vascular density, and the distribution of polarized M2 macrophages. In the absence, or inhibition of Gal-3, inflammation was reduced [26].

**Glucose oxidase loaded into bovine serum albumin nanoparticles** (BSA-Gox-NP) and injected intraperitoneally in a mouse model of EM acted as a non-hormonal therapy without side effects. The intraperitoneal injection of these neutrophil hitchhiking albumin nanoparticles resulted in a high specific enrichment in ectopic lesions in a neutrophil-dependent manner. Glucose oxidase was able to counteract the higher glucose uptake observed in endometrial stromal cells from patients with EM (eESC) compared to those from individuals without EM (euESC) [27].

**Table 1:** Potential immunotherapy candidates for EM treatment.

Drug Name and Structure	Target	Model	Effects	Reference
IL-33 Ab	IL-33	Women (*n* = 26) In vitro ESC Mice In vivo 50 μg + Erastin 300 μL 10 days	↓Development EM ↓Ferropoptosis tolerance eESC ↑M1 macrophage polarization	[13]
IL-8 Ab AMY109	IL-8	Monkeys In vivo 2 or 10 mg/Kg/4 weeks subcutaneous 6 months	↓Nodular lesions, ↓volume adhesions, neutrophils migration inhibition	[14]
IL-6R Ab Tocilizumab	IL-6R	Rats In vivo 8 mg/Kg/2 tw Intraperitoneally 4 weeks	Suppress volume endometriosic lesions	[15]
CTLA-4 Ab	CTLA-4	Mice In vitro peritoneal fluid 0.1 mg/mL 14 days	Proliferation and invasion of ectopic endometrial cells suppression	[17]
CD47 Ab siRNA	CD47	Women (*n* = 23) (10 EAOC, 13 eESC) In vitro Mice In vivo Intraperitoneal 0, 0.5, 2 and 4 µg/mL 12 h	Inhibition of growth and cellular migration, ↑apoptosis	[19]
CD20 Ab Rituximab	CD20	Rats In vivo 10 mg/Kg Intraperitoneal 14 days	↓Endometriotic implant size	[20]
EP4 antagonist	PGE2R	Women (*n* = 35) In vitro Normal uterus tissues, ovarian EM tissues, adenomyosis tissues and peritoneal fluid	↓cAMP levels, ↓expression growth factor	[21]
Lipoic acid	cAMP, protein kinase A signaling/ NF-kB	Women In vitro ESC 1–10 mM	↓NALP-3 ↓IL-1β, IL-18 ↓ICAM-1, ↓activity MMP2, 9 ↓invasion	[23]
TGR5 agonist INT-777	TGR5	Women In vitro ESC 5 µM/24 h 10 µM/48 h	Agonist, ↓proinflammatory cytokines, ↓adhesion molecules, Inhibits NF-κB	[24]
Sunitinib	p-VEGFR-PI3K-AKT-YBX1-Snail signalling pathway	Women (*n* = 3) In vitro eESC 0.1 µM 24 h	Receptors inhibitor, Kinases inhibition, ↓tumor migration, ↓tumor invasion	[25]
Gal3C	Galectin3	Mice In vivo 50 µg/day Intraperitoneal 15 days	↓Implantation, ↓size of lesions, ↓expression of VEGF and VEGFR-2, ↓inflammation, ↓COX-2, ↓TGFβ1	[26]
BSA-GOx-NP	Neutrophils	Mice In vivo 5 μL 10 mg/mL Intraperitoneal 14 days	↑Neutrophils	[27]

Ab antibody; EAOC EM-associated ovarian cancer cells; ESC endometrial stromal cells; ↓ decreases; ↑ increases or favours; *n* number of women affected with EM participating in the trial; tw times week. When possible, drugs were hyperlinked, to corresponding entries in https://www.guidetopharmacology.org, accessed on 30 May 2024.

## 3. Natural Compounds with Antioxidant and Anti-Inflammatory Activity

Related to the treatment of endometriosis, we can distinguish between drugs that can serve as immunotherapy and natural compounds with anti-inflammatory and antioxidant properties. Immunotherapy focuses on modulating the immune system to specifically target the underlying pathological processes in endometriosis. This includes the use of biological agents that can alter the immune response and reduce the proliferation of ectopic endometrial tissue. On the other hand, natural compounds, although they can also influence inflammation and oxidative stress, tend to act more broadly and less specifically. Their main benefit lies in reducing inflammatory symptoms and oxidative stress, which are significant factors in the pathogenesis of endometriosis, but they do not directly address the specific immunological abnormalities of the disease.

It is important to note that some drugs could be categorized under both immunotherapy and natural anti-inflammatory/antioxidant compounds. Additionally, we have considered the natural origin of products in our classifications. Moreover, natural products available in foods considered as nutraceuticals are regulated by different legislation compared to pharmaceutical drugs. This differentiation justifies placing them in separate tables, allowing for better analysis and leveraging the strengths of each type of intervention.

Some natural compounds that can suppress free radicals may be effective in endometriosis-related pain [28]. The antioxidant and anti-inflammatory natural compounds under study for EM and associated pain progression treatment are summarized in Table 2.

In traditional Chinese medicine, **Gui Zhi Fu Ling Wan** herbs mixture (Cinnamon, Poria, Safflower, Tree peony, Peony and Red sage) has been used for centuries for gynaecological disorders. Its decoction relieves primary menalgia and reduces uterine contractions by 55%. These encapsulated herbs decreased nitric oxide, reduced oxytocin-induced responses and prostaglandinF2α (PGF2α) in a trial with mouse models. It was concluded that this extract could restrict intracellular calcium levels [29]. The decoction extract has recently been shown to inhibit inflammation mediated by the PI3K/Akt signalling pathway in rats, among other actions [30]. A randomized, double-blind trial was conducted with Gynoclear^®^, a modification of Gui Zhi Fu Ling Wan herbs (two capsules/3 times a day for 12 months). Improvements in QoL, dyspareunia and fatigue were registered, as well as a reduced need for rescue analgesics [31].

**Sulforaphane** is an isothiocyanate isolated from cruciferous vegetables with anti-inflammatory and pro-apoptotic properties that has been tested in EM rat model. Sulforaphane intragastrical administration (from 5 to 30 mg/kg) for 3 weeks decreased dose-dependently the size of endometriotic foci and adhesion score, as well as the levels of IL-6, IL-10, TNF-α, IFN-γ, and VEGF in peritoneal fluid and plasma. It also regulated the apoptosis-related biomarkers including Bcl-2, Bax, and cleaved caspase-3. This effect was mediated by inhibition of PI3K/Akt signaling pathway [32]. Other work demonstrated that sulforaphane (5, 15, 30 and 60 mg/kg/day intraperitoneal for 28 days) inhibited ectopic endometrial tissue growth and alleviated pain induced by sciatic EM in rat EM model, mediated by inhibiting inflammation through Keap1 and Nrf2 pathway upregulation [33].

**Danefukang extract** contains Panax pseudoginseng and Corydalis. A study comparing the effect of mifepristone (at an initial dose of 12.5 mg, followed by 6.25 mg once daily for 10 days) and 15 g of danefukang extract (twice daily starting 15 days before the menstrual cycle) for three menstrual cycles found an effectiveness rate of 93.10% and 81.61% respectively. Danefukang treatment improved symptoms, QoL, depression, and anxiety, and reduced the levels of tumour necrosis factor alpha (TNF-α), IL-6, and CA-125 [34].

**Fisetin** is a flavonol (3,3′,4′,7-tetrahydroxyflavone) found in fruits and vegetables. The therapeutic target could be the pyrin domain of inflammasome receptor 3 (NLRP3) family. Fisetin oral administration (40 mg/kg on day 7 and the following 7 days) reduced the endometriotic implantation in an experimental rat model of EM, as well as neutrophil infiltration, cytokine release, mast cell numbers along with chymase and tryptase expression, and decreased expression of smooth muscle actin and transforming growth factor beta. In addition, fisetin reduced oxidative stress, as well as nitro tyrosine and poly ADP ribose expression, and increased apoptosis in endometrial lesions [35].

**Quince seed mucilage** is a mixture of water-soluble polysaccharides and cellulose. Two cc of quince seed plus 50 mg/kg/day hesperidin have been administered vaginally in an EM experimental model with rats for 21 days. Treatment restored the pathology and showed protective effects in uterine tissues. It reduced the MAPK expression and inflammation by decreasing the TNF-α levels [36].

In vitro studies have been carried out with different formulations of **Copaiba oil** (COPA) in endometrial stromal cell cultures from endometriotic lesions compared with the endometrium of patients without EM. This oil is rich in **β-caryophyllene,** which is a phytocannabinoid not derived from cannabis. The results showed a decrease in cell viability and morphological modifications in endometrial cells at concentrations higher than 150 μg/mL of nanoemulsions loaded with COPA for 48 h [37].

Kampo medicine, **TSS** (tokishakuyakusan), has shown anti-inflammatory and antiangiogenic effects on EM. Samples of endometriotic tissues and peritoneal macrophages were obtained for this study. In parallel, a pharmacokinetic study of **ferulic acid**, the active component of TSS, was carried out in rats. This trial demonstrated that ferulic acid decreased the secretion of IL-8 and angiogenic factor (VEGF) in endometrial stromal cells (ESC). It also suppressed the secretion of IL-8 by macrophages in the peritoneal fluid [38].

In other study, eutopic and ectopic ESC (euESC and eESC) samples were obtained from patients with EM, as well as euESC from controls without EM. This study found that treatment with 100 μmol/L of **resveratrol** (trans-3,5,4′-trihydroxystilbene), a natural phenolic compound found in certain plants, induced a significant reduction in gene and protein expression of MCP-1, IL-6 and RANTES (regulated on activation, normal T cell expressed and secreted) in both, euESC and eESC. This reduction was more notable in the ectopic than in the eutopic cells [39].

**Baicalein**, a natural flavonoid derived from the Chinese medicinal herb Huang-Qin or Scutellaria, has shown a variety of biological effects, including antioxidant, anti-inflammatory, and anti-cancer properties. Ferroptosis is a programmed cell death process characterized by iron-dependent accumulation of reactive oxygen species and lipid peroxidation [40]. From the review by Ni and Li [41], three key conclusions about ferroptosis emerge: first, excessive iron concentrations in EM patients, attributed to retrograde menstruation, induce oxidative stress and lipid peroxidation. Second, ferroptosis as a cell death pathway highlights cellular damage caused by lipid peroxidation, with endometriotic cells increasing resistance to ferroptosis through multiple mechanisms, allowing them to implant and proliferate in the peritoneal cavity. Third, the application of appropriate antioxidants in this study mitigated the toxic effects of reactive oxygen species generated by iron metabolism. This iron overload directly impacts the severity of endometriotic lesions, infertility, symptom severity, and malignancy. It has recently been highlighted that ectopic endometrial tissues resist iron overload-induced ferroptosis, promoting the growth of the ectopic lesions. Baicalein has been investigated as a potential antiferroptotic agent in women with EM. It suppressed ferroptosis-mediated inhibition of phagocytosis, particularly in patients with high ferroptosis and low phagocytic activity of macrophages [42].

**1,25 dihydroxycholecalciferol** (1,25 (OH)2 D3) is the active form of vitamin D3 and interacts with its nuclear receptor VDR. In a study conducted on endometrial tissue (*n* = 15), 1,25(OH)2D3 significantly counteracted LPS (lipopolysaccharide)-induced TNF-α production by ESCs and IL-6 production by whole endometrial cells (WECs). It also reduced TNF-α and IL-6 production in LTA-stimulated ESCs, decreasing inflammatory cytokine production by monocytes. Additionally, it increased IL-8 production in WECs and ESCs. The LPS-induced upregulation of MyD88 gene expression in WECs was normalized when these cells were pre-treated with 1,25(OH)2D3. The downregulation of MyD88 expression could inhibit TLR2 and TLR4 signaling, leading to decreased levels of proinflammatory cytokine production [43].

**Ascorbic acid**. A trial was conducted with 60 women who had laparoscopically confirmed endometriosis stages I-III. They were divided into two groups of 30 participants each. Group A received a combination therapy of 1000 mg of vitamin C plus 800 IU of vitamin E per day, while group B received a placebo. After 8 weeks, malondialdehyde and reactive oxygen species (ROS) levels in the blood had significantly reduced in the vitamin-treated group, indicating improved oxidative stress. Additionally, intergroup comparison showed a significantly greater reduction in dysmenorrhea, dyspareunia, and chronic pelvic pain in group A than in group B, with no adverse effects were described [44].

**Omega fatty acids**. An in vivo study in rat model of EM, the effect of omega fatty acids was analyzed and compared with medroxyprogesterone acetate (progestin) and meloxicam (NSAID). It was observed that omega-6/3 and omega-9/6 combinations decreased pain compared to the controls [45]. Subsequently, the pilot trial PurFECT1 was published, which studied the effectiveness of Omega- 3 polyunsaturated fatty acids. In this trial, conducted in 33 women, 17 took a 1000 mg capsule twice a day and 16 took an olive oil capsule twice a day for 8 weeks. Although the results were not statistically significant, there was a trend toward improvement in pelvic pain and quality of life scores in both treatment groups for the duration of the intervention. Therefore, the investigators see feasibility of conducting this study on a larger scale [46], with no adverse effects described in any of the tests.

**Table 2:** Proposed antioxidant and anti-inflammatory natural compounds for EM treatment.

Drug Name and Structure	Target	Model/Dose	Effects	Reference
Gui Zhi Fu Ling Wan (GZFLW)	PI3K/Akt pathway	Mice In vivo 0.54–1.08 g/Kg oral	↓Uterine contractions ↓NO, ↓PGF2α, ↓Ca^2+^	[29]
		Rats In vivo 1.89 g/mL	Inhibits inflammation, ↓volume of lesions, ↓sensitivity to pain	[30]
		Women (*n* = 90) In vivo 2 caps Gynoclear© /3 td oral 12 months	↓Dyspareunia, ↓fatigue, ↑QoL	[31]
Sulphoraphane	PI3K/Akt pathway Nrf2/ARE pathway	Rats In vivo 5–30 mg/Kg intragastrical 3 weeks	↓Endometriotic foci, ↓adhesion score, ↓ IL-6, IL-10, TNF-α, IFN-γ, and VEGF	[32]
		Rats In vivo 5,15,30,60 mg/Kg/day intraperitoneal 28 days	Inhibit ectopic endometrial tissue growth, ↓pain	[33]
Danefukang extrac (DEFK)	Not described	Women (*n* = 174) In vivo 15 g/2td/15 days Mifepristone 12.5 mg–6.25 mg/day/10 days 3 months	↑QoL, ↓depression, ↓anxiety, ↓TNF-α, ↓IL-6, ↓Ca125	[34]
Fisetin	NLRP3	Rats In vivo 40 mg/kg/day oral 14 days	↓Endometriotic implantation, ↓mast cell infiltration, ↓fibrosis, ↓histological alterations, ↓neutrophil infiltration, ↓cytokine release, ↓oxidative stress, ↓nitrotyrosine, ↓poly ADP ribose expressions, ↑apoptosis in endometrial lesions	[35]
Quince seed Mucilage	MAPK pathway Caspase-3	Rats In vivo 2 cc/day vaginal +50 mg/kg/day Hesperidin 21 days	↓Inflammation, ↓apoptotic process, ↓mitochondrial damage, restoring the uterine mucosa	[36]
Ferulic acid	Not described	Women (*n* = 27) In vitro ESC and peritoneal macrophages. 500 µM Rats In vivo 4 g/Kg once oral 2 h	↓Inflammatory cytokines, ↓angiogenic factor (VEGF)	[38]
Resveratrol	MAPK pathway	Women (*n* = 22) In vitro *ESC * 100 µmol/L	↓MCP-1, ↓IL-6, ↓IL-8, ↓RANTES	[39]
Baicalein	MAPK pathway Nrf2/ARE pathway	Women (*n* = 8) In vitro 20 µM ectopic endometrium, peritoneal and cyst fluid	Ferroptosis inhibition Restauration phagocytosis	[42]
1,25 (OH)2 D3	VDR	Women (*n* = 0) ESC and WEC In vitro	↓TNF-α, ↓inflammatory cytokines	[43]
Ascorbic acid	Not described	Women (*n* = 60) In vivo 1000 mg + 800 IU vitamin E/day oral 8 weeks	↓malondialdehyde, ↓ROS, ↓dysmenorrhea, ↓dyspareunia, ↓CPP	[44]
Omega fatty acids	Not described	Women (*n* = 30) In vivo 1000 mg/2 td 8 weeks	↓PP, ↑QoL	[46]

Caps capsules; td times a day; ↓ decreases; ↑ increases, favors; QoL quality of life; ESC Endometrial stromal cells; CPP chronic pelvic pain; 2td twice a day; PP pelvic pain; *n* number of women affected with EM participating in the trial. When possible, drugs were hyperlinked to corresponding entries in https://www.guidetopharmacology.org, accessed on 30 May 2024.

## 4. Repurposed Drugs

Repurposing drugs presents a streamlined pathway to innovative therapies by leveraging existing medications for new medical indications. This approach capitalizes on the established safety profiles and known pharmacological properties of these drugs, potentially accelerating the development process and expanding treatment options for various medical conditions. Repurposed drugs under study for EM and associated pain treatment are summarizes in Table 3.

A non-randomized prospective observational study analyzed a treatment with the antibiotic **levofloxacin** (500 mg orally 24h before surgery), alone or in combination with GnRHa (1.88 mg/month intramuscularly, for 3 months before surgery) in EM patients and women without EM as control group. Treatment with levofloxacin and/or GnRHa induced a decrease in tissue inflammation, cell proliferation, and angiogenesis in the endometrium and endometriotic lesions with histologically proven improvement of ovarian endometrioma. It could improve lesion recurrence and pain in women with EM [47].

The antifungal agent **clotrimazole** decreased the growth of endometriotic lesions in an EM rat model at 200 mg/kg for 15 consecutive days. An improvement in the antioxidant system and a significant decrease in the distribution of inducible nitric oxide synthase (iNOS) in the lesions, as well as in the levels of lipid peroxidation and protein carbonylation (biomarkers of oxidative stress) in the liver, were also detected. Therefore, clotrimazole prevents cell damage and inflammatory processes [48].

**Loratadine**, an H1-antihistamine, acts as an antagonist of the transient receptor potential cation channel subfamily V member 2 (TRPV2), a calcium-permeable cation channel. An in vitro study proposed loratadine as a potential treatment for EM. It inhibited TRPV2-dependent responses in a primary culture of mouse endometrial stromal cells and reduced cell proliferation, migration, and wound healing in assays [49].

**Quinagolide**, a non-ergot dopamine D2 receptor agonist used in treating hyperprolactinemia, has shown significant efficacy in regression of endometriotic implants and significantly reducing levels of IL-6 and VEGF in peritoneal fluid in a rat model of EM [50]. In endometrial mesenchymal stromal cells obtained from women with stage III or IV EM, quinagolide induced a reduction of endothelial invasion and differentiation through the AKT signalling pathway, negatively regulating AKT and its phosphorylation [51].

A trial with **cabergoline**, another D2 receptor agonist, was performed in patients with confirmed genital EM (grade I-III) and a control group of 12 women without gynaecological pathology over six months. EM patients received cabergoline (0.25 or 0.5 mg twice weekly) alone or combined with GnRHa or dienogest (2 mg), with results compared to GnRHa and dienogest monotherapy (2 mg). The combination of cabergoline with standard hormone therapy regimens had a more pronounced effect on pain syndrome and can also be used as monotherapy for patients with contraindications to hormone therapy [52].

**N-acetylcysteine**, commonly used to reduce the thickness of mucus and facilitate its removal, has shown an antioxidant activity upstream of the COX-2 pathway. A prospective cohort study evaluated its efficacy in women with EM who were not undergoing hormonal treatment or pregnancy. The study found significant reductions in ovarian endometrioma size, serum Ca125 levels, dysmenorrhea intensity, dyspareunia, and chronic pelvic pain. Additionally, the therapy appeared to improve fertility, with 39 out of 52 patients achieving pregnancy within six months [53].

**Niclosamide**, an anthelmintic drug administered at 200 mg/kg/day orally for three weeks, reversed the transcriptomic changes in macrophages caused by lesion induction in a mouse model of EM after six weeks. This effect was achieved through its regulation of the differentiation of recruited macrophages and the maturation of transient large peritoneal macrophages (LPM). Niclosamide also restored the disrupted communications between resident LPM and B cells caused by injury induction [54].

## 5. Conclusions and Perspectives

Current management options for EM include surgical and medical treatments. The efficacy of surgery for EM-associated PP treatment is compromised by postoperative recurrence [10], and some interventions pose risks, such as diminished ovarian reserve and heightened complications [11]. Hormone therapy, targeting the estrogen-dependent nature of this disease, is often initiated in suspected cases before surgical confirmation and continued post-surgery for persistent symptoms [9]. Commonly prescribed treatments for EM include drugs that alter the hormonal milieu, suppress ovarian activity, or act directly on steroid receptors and enzymes found in the lesions. Although various hormonal treatments exist, they exhibit similar efficacy in pain reduction but differ in their side effect profiles. Systemic side effects often lead to non-adherence and discontinuation of therapy, particularly challenging for long-term use [9].

Additionally, the patient’s desire for pregnancy must be considered due to the contraceptive properties of these treatments. When side effects or poor tolerability force the interruption of hormone therapy, pain and lesions often reappear quickly [7]. We have demonstrated promising outcomes through innovative interventions involving immunotherapy, natural anti-inflammatory agents and antioxidants, and repurposed drugs. They could offer a non-invasive solution to the pain and infertility associated with EM, providing benefits not offered by standard hormonal drugs, which neither improve fertility nor can be used by patients trying to conceive. The potential for clinical translation in the treatment of endometriosis is promising, yet each approach presents unique challenges and opportunities. Repositioned drugs offer known safety profiles and quicker regulatory approval. They can effectively manage symptoms and modify disease progression. However, the complex pathology of endometriosis means not all repositioned drugs will be effective, necessitating clinical trials to determine their efficacy. Natural antioxidants and anti-Inflammatory compounds, including nutraceuticals, provide a safer approach to managing endometriosis by reducing oxidative stress and inflammation. However, their variable bioavailability and potency require more research for standardization. Different regulatory frameworks for nutraceuticals can impact their clinical application. Immunotherapy targets specific immune pathways involved in endometriosis, offering significant potential. The challenge is precise targeting to avoid broad immunosuppression and adverse effects. Extensive clinical trials are needed to establish safety and efficacy.

Each approach has its benefits and limitations. Repositioned drugs are fast-tracked but may not address specific mechanisms. Natural agents are safe and accessible but need validation. Immunotherapy is targeted but requires thorough research. Combining these strategies, tailored to individual patient profiles, could offer comprehensive and effective management of endometriosis.

These approaches have primarily been investigated in vitro and in vivo using animal models, indicating emerging potential.

Investigations utilizing both in vivo and in vitro models for EM have expanded our knowledge, though they face certain constraints in their exploration. Results found in animal models often do not translate to humans. This is the case of recombinant human TNF-α binding protein, whose positive results in rat and baboon model were not paralleled on human [55]. New and emerging in vitro models, such as organoids and microfluidics, provide a new frontier for studying endometrial diseases such as EM [56].

It has been identified two subgroups of EM using gene expression data and tissue microarrays, associated with the failure of hormone therapy [57]. It also has been described three subphenotype-specific cytokine signatures, describing EM better than disease stages [58]. These results showed that EM is not one disease, and classify patients based on molecular profiling to guide treatment could improve therapeutic results.

Further research is needed to translate these promising results into clinical practice, potentially offering new therapeutic avenues to patients, more personalized and effectives.

## Figures and Tables

**Table 3:** Repurposed drugs in study for EM and associated pain treatment.

Drug Name and Structure	Target	Model/Dose	Effects	Reference
Levofloxacin 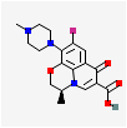	Bacterial topoisomerase IV and DNA gyrase	Women (*n* = 53) In vivo 500 mg/24 post-surgery/oral or +1.88 mg GnRHa/month intramuscular 3 months	Inhibition bacterial topoisomerase IV and DNA gyrase, ↓tissue inflammation, ↓cell proliferation, ↓angiogenesis endometriotic, ↓lesions pain	[47]
Clotrimazole 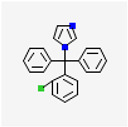	CYP53 enzyme	Rats In vivo 200 mg/kg oral 15 days	CYP53 enzyme inhibition, ↓inflammation, ↓endometric lesions, ↓distribution of iNOS, ↑antioxidant system	[48]
Loratadine 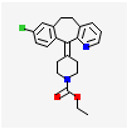	TRPV2	Women In vitro Cell culture ESC	Antagonist, ↓cell proliferation/ migration, ↓inflammation	[49]
Quinagolide 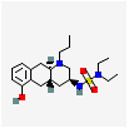	Dopamine D2 receptor	Rats In vivo 200 µg/Kg/day oral 4 weeks	↓ Endometriotic implants, ↓IL-6, ↓VEGF	[50]
		Women (*n* = 10) In vitro ESC 24 h	Agonist, ↓lesion size, ↓invasion and differentiation, ↓AKT signaling pathway	[51]
Cabergoline 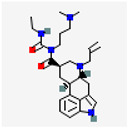	Dopamine D2 receptor	Women (*n* = 227) In vivo 0.25–0.5 mg/twice/week oral cabergoline + hormone therapy 6 months	↓Pain syndrome	[52]
N-acetyl-L-cysteine 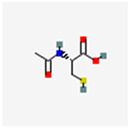	COX2 pathway	Women (*n* = 120) In vivo 600 mg/day/3 consecutive days/ week oral 3 months	↓Dysmenorrhea, ↓dyspareunia, ↓CPP, ↓endometrioma size, ↓serum Ca125 levels, improve fertility	[53]
Niclosamide 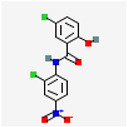	Wnt/β-catenin signal pathway Macrophage activity	Mice In vivo 200 mg/Kg/day/3 weeks oral 6 weeks	Reverse macrophage transcriptomic changes in EM Rescue LPM and B cell communication after EM induction	[54]

↓ decreases; ↑ increases, favors; CPP chronic pelvic pain; NMPP non menstrual pelvic pain, ESC Endometrial stromal cells; TRPV2 transient receptor potential cation channel subfamily V member 2; HMG-CoA 3-hidroxi-3-metil-glutaril-CoA; *n* number of women affected with EM participating in the trial. When possible, drugs were hyperlinked to corresponding entries in https://www.guidetopharmacology.org, accessed on 30 May 2024.
